# Can AI-Predicted Complexes Teach Machine Learning to Compute Drug Binding Affinity?

**DOI:** 10.1021/acs.jcim.5c01848

**Published:** 2025-12-10

**Authors:** Wei-Tse Hsu, Savva Grevtsev, Anna M. Herz, Thomas Douglas, Aniket Magarkar, Philip C. Biggin

**Affiliations:** † Structural Bioinformatics and Computational Biochemistry, Department of Biochemistry, 6396 University of Oxford, South Parks Road, Oxford, OX1 3QU, U.K.; ‡ Department of Chemistry, University of Oxford, Mansfield Road, Oxford, OX1 3TA, U.K.; § 33437 Boehringer Ingelheim Pharma GmbH & Co. KG, Birkendorfer Str. 65, 88397 Biberach an der Riß, Germany

## Abstract

We evaluate the feasibility of using co-folding models for synthetic data augmentation in training machine learning-based scoring functions (MLSFs) for binding affinity prediction. Our results show that performance gains depend critically on the structural quality of augmented data. In light of this, we established simple heuristics for identifying high-quality co-folding predictions without reference structures, enabling them to substitute for experimental structures in MLSF training. Our study informs future data augmentation strategies based on co-folding models.

## Graphical abstract

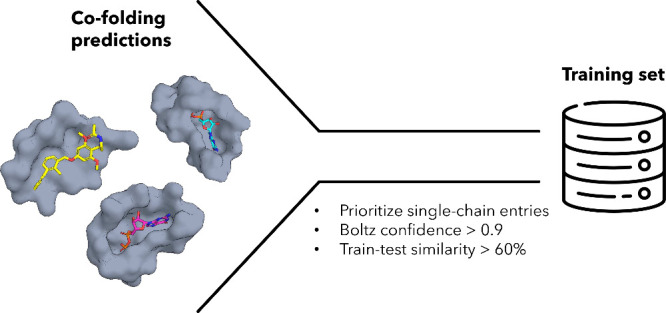

## Introduction

Over the past decades, machine learning-based scoring functions (MLSFs) have gained increasing popularity in computer-aided drug discovery.^1^ By leveraging 3D structures of binding complexes—usually protein-ligand binding complexes—these models predict binding affinities in a fraction of the time required by physics-based simulation methods such as alchemical free energy perturbation,^2^ while achieving arguably comparable accuracy in some scenarios.^3^ During training, they often rely on experimental structures of binding complexes, representing binding interfaces with underlying architectures ranging from feed-forward neural networks,^4^ convolutional neural networks (CNNs),^5^ transformers,^6^ to graph neural networks (GNNs).^3,7^ However, the data of high-resolution experimental complexes with matched binding affinity measurements remain rare, limiting both the scale and diversity of training data sets available for these models.

To address this scarcity, several efforts have emerged to synthetically augment training data sets using computational modeling. One notable example is BindingNet,^8^ which uses protein structures from PDBbind^9^ as templates and models new complexes by aligning structurally similar ChEMBL^10^ ligands to the reference ligands based on their maximum common substructures. With this template-based modeling approach, BindingNet v1 generated approximately 70K protein-ligand complexes with associated activity data from ChEMBL. Its successor, BindingNet v2,^11^ introduced a hierarchical variation of the modeling pipeline to accommodate less similar candidate ligands, further expanding the data set to roughly 700 K complexes. Recent studies have demonstrated that the inclusion of BindingNet v1 improves the performance of MLSFs,^3^ though BindingNet v2 has so far only been used to train the docking model Uni-Mol,^12^ where improved success rates in PoseBusters^13^ sanity checks were observed, i.e. more physical binding poses were generated.

One inherent drawback of these template-based modeling approaches, however, is their reliance on high-quality, experimentally determined protein structures as templates, which restricts the extent of data augmentation. Moreover, these methods implicitly assume that structurally similar ligands that bind to the same protein receptor share the same binding mode, which does not always hold in practice. Recent advances in co-folding models, such as AlphaFold3 (AF3),^14^ Chai-1,^15^ and Boltz,^16,17^ enable *de novo* structure prediction of protein-ligand complex structures, offering a promising alternative to further expand the scope of binding complex data sets. Indeed, a large-scale data set generated using Boltz-1x has been recently proposed by Lemos et al.^18^ Yet, the use of co-folding predictions for large-scale data set generation has not been systematically examined in the context of training MLSFs.

In this work, we report key insights from training two state-of-the-art GNN-based models (AEV-PLIG^3^ and EHIGN^19^) and one random forest-based model (RF-Score^20^) on multiple modeled complex data sets, including BindingNet v1, BindingNet v2, and Boltz-1x-based reproductions of a recently introduced experimental data set HiQBind.^21^ Our study focuses on three fundamental questions: (1) To what extent does data augmentation improve MLSF performance, especially when introduced with synthetic training examples that are not necessarily of high quality? (2) Are co-folding predictions sufficient to replace or complement experimental structures for MLSF training? (3) What practical heuristics can be used to identify high-quality co-folding predictions in the absence of reference structures? By systematically addressing these questions, we aim to provide early but essential insights into how best to leverage co-folding models in large-scale data set construction for structure-based machine learning.

## Results and Discussion

### Data Augmentation Benefits Can Be Diluted by Low-Quality Examples

We first assessed the impact of synthetic data augmentation on the performance of machine learning-based scoring functions. Figure 1A presents the performance of AEV-PLIGs trained on different combinations of HiQBind, BindingNet version 1, and v2. Performance was evaluated using Pearson correlation coefficient (PCC) for scoring power and Kendall’s τ for ranking power, both computed between the predicted and experimentally measured binding affinities on the FEP benchmark data set,^22^ a challenging test set with minimal data leakage from our training sets (see Figure S5). As a result, the addition of BindingNet v1 substantially improved model performance, consistent with previous findings.^3^ Interestingly, the inclusion of BindingNet v2 (despite an increase in the training set size by over 7-fold) did not yield any further noticeable improvement.

**Figure 1:**
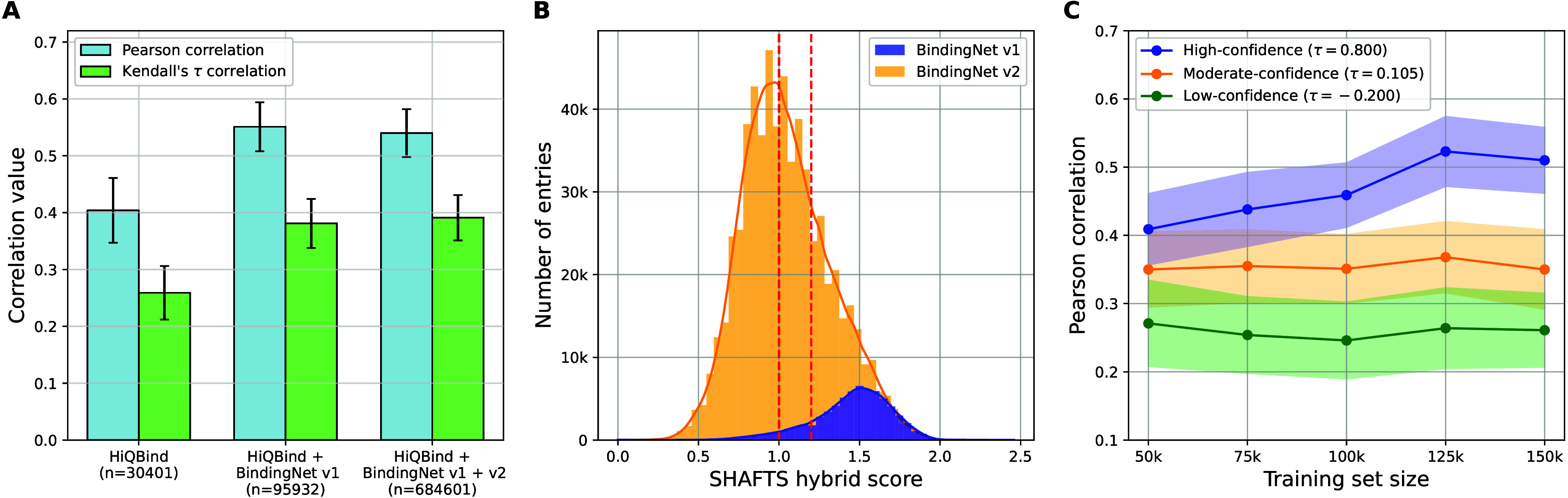
Performance of AEV-PLIGs trained on various combinations and partitions of HiQBind, BindingNet v1, and BindingNet v2. (A) Model performance when trained on HiQBind alone, HiQBind + BindingNet v1, and HiQBind + BindingNet v1 + BindingNet v2. The sizes of the data sets are noted in parentheses in the labels. (B) Distribution of SHAFTS hybrid scores in BindingNet v1 and v2, with two vertical lines marking the cutoffs for different confidence partitions. (C) Performance of models trained on progressively larger subsets of BindingNet v1 + v2, constructed from different confidence partitions. Each larger subset includes all smaller ones. The Kendall’s τ correlations between PCC and training set size for different cases are annotated in the legend.

To gain more insights into this, we examined the SHAFTS^23^ hybrid score, a confidence metric used in BindingNet. According to the original BindingNet v2 study,^11^ structures are categorized as high-confidence if their hybrid score exceeds 1.2, moderate-confidence if between 1.0 and 1.2, and low-confidence if below 1.0. As shown in Figure 1B, most entries in BindingNet v2 fall into the low- to moderate-confidence range, whereas BindingNet v1 exhibits a markedly higher median confidence score (1.48 versus 1.02). The original study^11^ reported a top-1 docking success rate (defined as a ligand RMSD < 2 Å) of only 16% for low-confidence structures, 33% for moderate-confidence, and 73% for high-confidence entries. The overrepresentation of lower-confidence entries in BindingNet v2 therefore strongly suggests that it contains a significantly higher proportion of low-quality structures than v1.

To quantify how such differences in data quality affect model performance, we trained AEV-PLIGs on subsets of the union of BindingNet versions 1 and 2 grouped by confidence levels. For each confidence partition, these subsets were constructed by successively introducing entries randomly drawn from the partition (Figure 1C). As a result, when models were trained solely on high-confidence structures, the model performance generally improved with an increasing training set size, although some suboptimal structures in the high-confidence subset may have tempered the overall gains. In contrast, no such improvement was observed for models trained on moderate- and low-confidence data. This is reflected by the Kendall’s τ correlation between PCC and training set size, which was 0.80 for high-confidence, but only 0.105 and –0.20 for moderate- and low-confidence subsets, respectively. Together, the results shown in Figures 1B and C help explain the negligible performance change upon inclusion of BindingNet v2 in the training set.

Importantly, the trends shown in Figures 1A and C are also observed in the same experiments performed with EHIGN (Figures S6A and B), a GNN model with a distinct architectural design. As for RF-Score (Figures S7A and B), we note that neither the inclusion of BindingNet v1 nor v2 improves the model performance, which is likely attributable to the model’s limited expressive power stemming from its lack of 3D geometry awareness. Overall, provided the scoring function has sufficient capacity to extract meaningful learning signals from training data, these findings demonstrate that data augmentation is effective for MLSF training only when the structural quality of newly introduced examples is sufficiently high. Simply adding more synthetic complexes without quality control offers limited benefit, highlighting the need for rigorous filtering in future data set construction efforts.

### Practical Heuristics Support Reliable Curation of Co-Folding Predictions

As the utility of synthetic data in MLSF training hinges on structural quality, one key challenge in data augmentation using co-folding models is to identify reliable predictions in the absence of reference structures. We therefore investigated whether simple heuristics could serve this purpose by analyzing Boltz-1x predictions on a series of structure reproduction tasks.

As a starting point, we used Boltz-1x^17^ to reproduce the recently introduced HiQBind data set,^21^ which is arguably the highest-quality experimental data set of protein-ligand complexes currently available for MLSF training. Boltz-1x, which is a variant of Boltz-1 with inference-time steering for generating physically plausible structures, has been shown to be among the strongest co-folding models for protein-ligand structure prediction,^27,28^ and is therefore an ideal choice for our task.

In the reproduction task, we replaced each experimental structure in HiQBind with its corresponding Boltz-1x prediction and then compared the prediction with the experimental reference to assess its quality. As illustrated in the Sankey diagram in Figure 2A, Boltz-1x exhibited greater confidence and performance when predicting complexes with single-chain receptors, consistent with past observations that mult-chain complex prediction is generally more challenging, usually due to weaker interchain coevolutionary signals, chain pairing ambiguity, and limited multichain training examples.^29,30^ Note that in Figure 2A, structures passing the PoseBusters^13^ sanity check are labeled “Physical”, those with a pocket RMSD below 2 Å with respect to the experimental references are lableled “High-quality”, and those with a Boltz confidence score above 0.9 are labeled “High-conf”, where the Boltz confidence score is defined as the sum of 0.8 × the complex pLDDT score and 0.2 × the iPTM score (or the pTM score for single-chain systems)^16^

**Figure 2:**
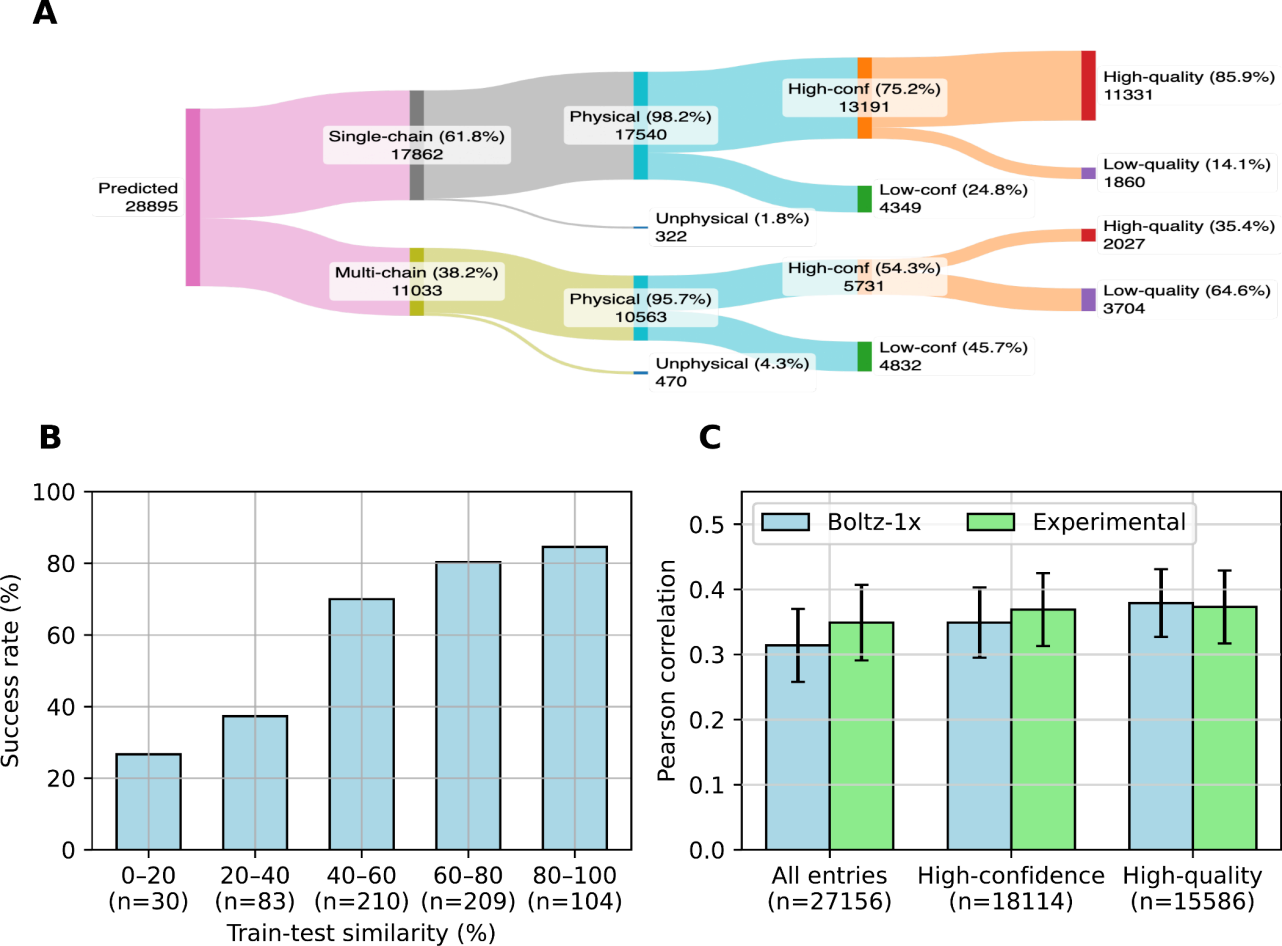
Results based on the HiQBind and RNP reproduction tasks. (A) Sankey diagram that summarizes the overall performance of Boltz-1x in the HiQBind reproduction task. Each flow is annotated with the number of predicted structures and its percentage relative to the preceding category. (B) Success rate (defined as the percentage of structures having a pocket RMSD < 2 Å) in the RNP reproduction task given different levels of train-test similarity defined in the RNP study,^24^ which is a product of binding pocket coverage^25^ and combined overlap score (SuCOS)^26^ of the ligand pose. (C) Performance of AEV-PLIGs trained on different subsets of HiQBind and their Boltz-1x-reproduced counterparts. The size of the training set in each case is annotated. Details about training set curation are available in the Methods section of the Supporting Information.

Upon examination of the confidence-quality relationship, we found that the Kendall’s τ correlation between commonly used confidence and quality metrics is generally weak (Figure S8), indicating that higher confidence does not necessarily imply higher structural quality. Still, for single-chain systems, a simple confidence threshold of 0.9 usefully identifies a subset in which 85.9% of predictions are high-quality (Figure 2A). This simple filtering strategy also applies to several other metrics. For example, Figure S9 shows that metrics like ligand pLDDT and interface pLDDT (ipLDDT) scores could also identify subsets in which at least 85% of entries are high-quality using thresholds at 0.62 and 0.75, respectively. At this enrichment level, these two metrics yield subsets of similar size to those obtained by filtering with Boltz confidence, suggesting comparable effectiveness (Figure S10). In contrast, the pTM score requires a much stricter threshold (0.95) and results in a noticeably smaller subset (Figure S10), and metrics such as PDE and PAE fail to identify any subsets meeting the 85% criterion (Figure S9).

Note, however, that HiQBind is within the training set of Boltz-1, which explains the overall decent performance observed in the reproduction task. Indeed, recent work by Škrinjar et al.^24^ demonstrated that the accuracy of Boltz-1 is largely dependent on the train-test similarity. This raises the important question of whether there exists a similarity cutoff above which we can still expect reasonable performance from Boltz-1x. To investigate this, we used Boltz-1x to reproduce the Runs and Poses (RNP) data set proposed by Škrinjar et al.,^24^ which comprises 2600 binding complex structures released after the training cutoff date of AF3-like co-folding models (including Boltz) and specifically designed to evaluate their generalizability. We focused on single-chain systems, which, as shown in Figure 2A, are more reliably predicted by Boltz-1x and therefore represent the most practical candidates for co-folding data augmentation. A Sankey diagram summarizing the basic statistics for the RNP reproduction task is shown in Figure S11.

As a result, Figure 2B shows that the success rate (defined as the percentage of predicted complexes having a pocket RMSD < 2 Å) declines with the train-test similarity, which agrees with the findings in the work by Škrinjar et al.^24^ Nonetheless, predictions with 60% to 80% train-test similarity still achieved a success rate of approximately 80%, suggesting that maintaining this level of similarity may serve as a practical guideline for prospective applications seeking reliable performance. Importantly, we observed that the confidence model in Boltz-1x appears more resistant to out-of-distribution shifts than the structure prediction module itself, as evidenced by the consistent confidence-quality correlation (Figure S12), and the relatively stable enrichment of high-quality predictions within high-confidence subsets across similarity bins (Figure S13). That is, even if high-confidence predictions may be less frequent for prospective campaigns targeting out-of-distribution domains, the confidence threshold heuristics should remain effective for identifying reliable structures when such predictions are available. Notably, given the architectural similarities and comparable performance reported across AF3-derived co-folding models,^16,24^ we expect the train-test similarity threshold to generalize. However, recalibrating the confidence threshold may be necessary when transferring the confidence heuristics, since confidence models may differ in their calibration across frameworks. Still, these findings collectively support the feasibility of applying simple heuristics to guide reliable co-folding-based data set construction at scale.

### Co-Folded Structures Support Scoring and Ranking in MLSF Training

To assess whether co-folding predictions can serve as a substitute for experimental structures in MLSF training, we compared the scoring and ranking power of AEV-PLIGs trained on different subsets of HiQBind and their Boltz-1x-reproduced counterparts. As shown in Figure 2C, AEV-PLIGs trained on Boltz-1x predictions achieved scoring performance statistically indistinguishable from those trained on the original experimental structures—whether using the full data set, only high-confidence predictions (Boltz-1x confidence score >0.9), or only high-quality predictions (pocket RMSD < 2 Å and validated by PoseBusters^13^). The same trends are also observed in the comparison of ranking power (see Figure S14A), suggesting that co-folded structures can provide training signals nearly equivalent to those of experimental structures for these tasks. Notably, including all Boltz-1x predictions, which led to a training set 74% larger than the high-quality subset, did not lead to improved performance. This again mirrors the observations in Figure 1A, reinforcing that data augmentation with low-quality examples offers little benefit in improving the scoring function performance. In the Supporting Information, we also show that these trends hold for EHIGN and RF-Score (Figures S6C, S7C, S14B, and S14C). Additionally, given the recent release of Boltz-2x^17^ near the completion of our work, we further confirmed in Figure S2 that Boltz-2x predictions can likewise support scoring and ranking in MLSF training. Given that Boltz-2 introduced the capability to perform binding affinity prediction, we also compared its performance with AEV-PLIG and FEP+^31^ on the FEP benchmark across various congeneric series for the community’s interest (see Figures S3 and S4).

Note, however, that the screening power of AEV-PLIGs trained on experimental structures and those trained on Boltz-1x predictions diverged markedly in a virtual screening task (Figure S15). This substantial gap likely arises from subtle but systematic structural differences in the training data that, while insufficient to affect affinity scoring or ranking, may reduce the models ability to distinguish true binders from nonbinders in large compound libraries. Further investigation will be required to clarify how the co-folding model biases contribute to this effect, and we provide methodological details about the enrichment experiments in the Supporting Information.

## Conclusions

High-quality binding structures are critical for training effective machine learning-based scoring functions, yet their scarcity remains a limiting factor. This study demonstrates that co-folding predictions, when properly filtered, may serve as viable substitutes for experimental structures in large-scale MLSF training, offering comparable scoring and ranking performance even when used as full replacements.

Through systematic evaluation, we established when synthetic augmentation improves model performance, how to reliably select useful co-folding predictions, and that filtered co-folded structures can match experimental ones in scoring and ranking assessments. In particular, we found that low-quality synthetic examples offer little benefit even when they dramatically increase training set size, underscoring the need for stringent quality control in data augmentation. We therefore further established practical heuristics, such as prioritizing single-chain complexes, filtering by Boltz-1x confidence score >0.9, and enforcing a train-test similarity above 60%, to effectively identify high-quality predictions in the absence of reference structures. These filtering strategies enable co-folding predictions to be used at scale without compromising model accuracy.

Taken together, our findings provide a practical foundation for extending MLSF data sets beyond experimentally determined structures, particularly in underrepresented protein families where structural data remain sparse. By enabling scalable, quality-controlled data augmentation, co-folding models hold promise for advancing the next generation of structure-based machine learning in drug discovery.

## Supplementary Material



## Data Availability

All AEV-PLIG experiments in this study were conducted using a refined version of the AEV-PLIG codebase, available under the 3-Clause BSD License: https://github.com/weitse-hsu/AEV-PLIG-refined and forked here: https://github.com/bigginlab/AEV-PLIG-refined. Specific splits used in different experiments can be found in a separate repository: https://github.com/weitse-hsu/AEV-PLIG-data and forked here: https://github.com/bigginlab/AEV-PLIG-data. Large-scale Boltz-1x predictions and subsequent analyses were performed using in-house code, which will be publicly released soon in a follow-up study.

## Notes

Supplementary analyses of AEV-PLIGs trained on different subsets of Boltz-2x-reproduced HiQBind. Methods section for elaborating methodological details of model training, testing, and reproduction tasks using Boltz-1x. Supplementary table of compound and active counts for the systems considered for the enrichment screening. Supplementary figures, including the distributions of maximum Tanimoto similarity between training set and test set ligands, performance of EHIGN and RF-Score in key experiments, correlation analysis on Boltz-1x-reproduced HiQBind, confidence-quality relationship examined in the HiQBind reproduction task, fraction of entries passing the threshold required to yield enriched subsets in the HiQBind reproduction task, Sankey diagram for the RNP reproduction task, distributions of Boltz confidence score for different train-test similarity levels, percentage of high-quality structures at different train-test similarity levels, and the performance of AEV-PLIG models in enrichment screening. (PDF)
