# Supplementary material for: Can AI-Predicted Complexes Teach Machine Learning to Compute Drug Binding Affinity?
Source: J Chem Inf Model. 2025 Dec 10;65(24):13051–6. doi: 10.1021/acs.jcim.5c01848 (PMC12728920; doi:10.1021/acs.jcim.5c01848)
Supplement: Supplementary file 1 [file ci5c01848_si_001.pdf]

Supporting Information: Can AI-predicted complexes teach machine learning to compute drug binding affinity?

Wei-Tse Hsu,[†] Savva Grevtsev,[‡] Anna M. Herz,[¶] Thomas Douglas,[†] Aniket Magarkar,[*,¶] and Philip C. Biggin[*,†]

†*Department of Biochemistry, University of Oxford, South Parks Road, Oxford, OX1 3QU, UK*

‡*Department of Chemistry, University of Oxford, Mansfield Road, Oxford, OX1 3TA, UK*

¶*Boehringer Ingelheim Pharma GmbH & Co. KG, Birkendorfer Str. 65, 88397 Biberach an der Riß, Germany*

E-mail: aniket.magarkar@boehringer-ingelheim.com; philip.biggin@bioch.ox.ac.uk

Supplementary analyses

AEV-PLIGs trained on Boltz-2x-reproduced HiQBind

In the main text, we showed that AEV-PLIGs trained on Boltz-1x predictions have similar performance to those trained on the corresponding experimental structures. Boltz-2x, which was released during the completion of our study, shares the same inference-time steering as Boltz-1x and adopts a similar architecture, and was reported to exhibit comparable performance in structure prediction for protein-ligand complexes[1] on a test set with a moderate out-of-distribution shift. Given these similarities, we hypothesised that AEV-PLIGs trained on Boltz-2x-reproduced structures would also match the performance of those trained on experimental data. As a supplementary analysis to confirm this expectation, we additionally used Boltz-2x to reproduce HiQBind and evaluated AEV-PLIGs trained on the reproduced dataset.

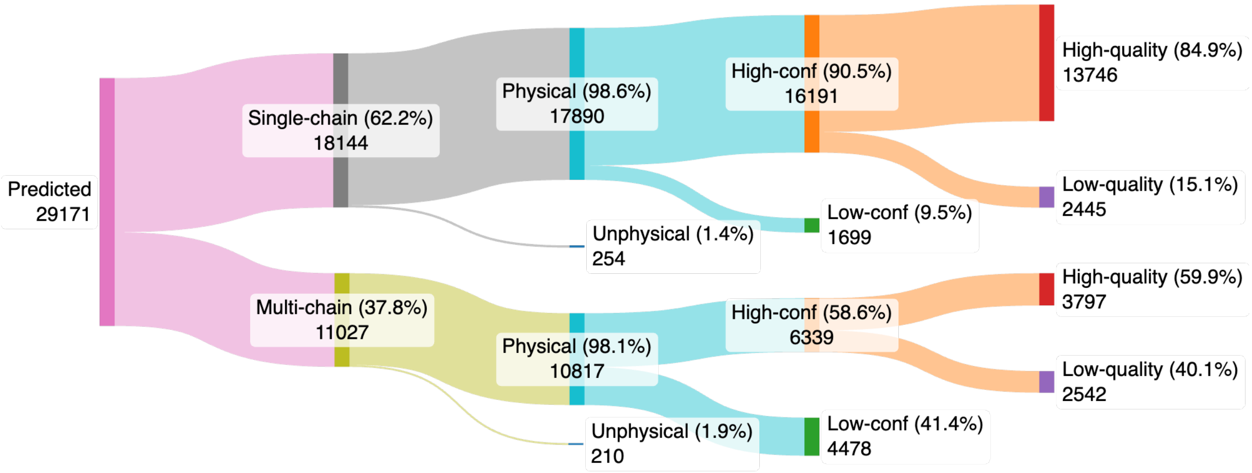

Figure S1: Sankey diagram that summarises the overall performance of Boltz-2x in the HiQBind reproduction task. Each flow is annotated with the number of predicted structures and its percentage relative to the preceding category.

As a result, the Sankey diagram in Figure S1 shows that Boltz-2x generated a larger number of high-quality structures for both single-chain and multi-chain structures, likely due to better alignment between HiQBind and Boltz-2's enhanced training data. Notably,

the confidence cutoff of 0.9 performs similarly to that in the Boltz-1x-reproduced HiQBind, identifying a subset in which 84.9% of the entries are of high quality, again demonstrating the generality of the heuristics developed in this work. More importantly, we show in Figure S2 that AEV-PLIGs trained on Boltz-2x predictions exhibited comparable scoring and ranking power as those trained on the corresponding experimental structures, validating that the Boltz-2x predictions are also sufficient to serve as training examples for MLSF in such tasks.

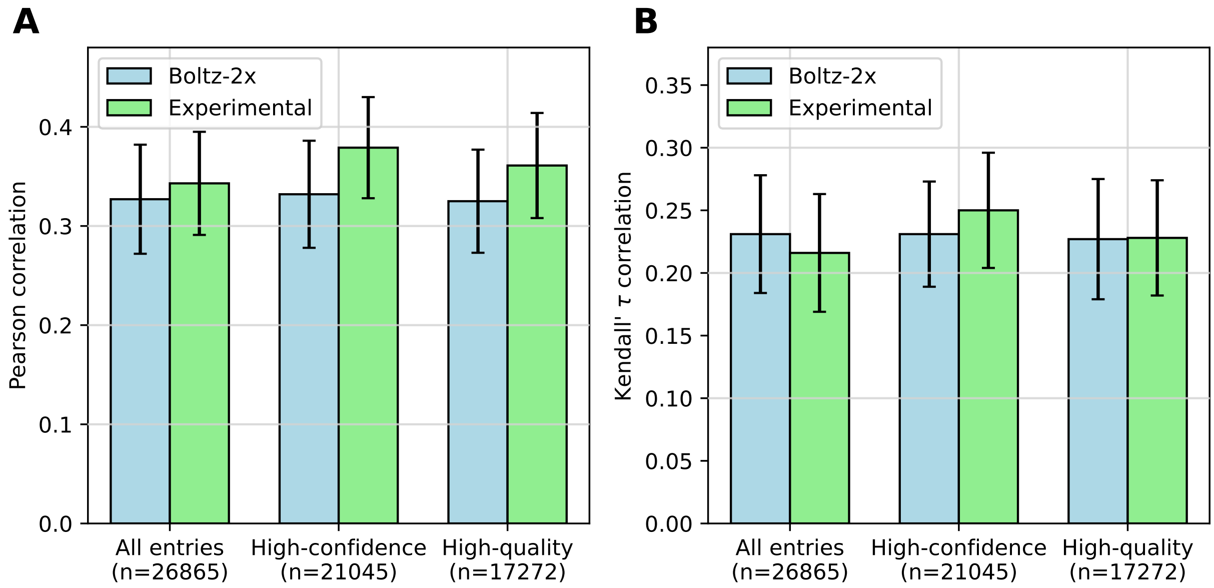

Figure S2: Performance of AEV-PLIGs trained on different subsets of HiQBind and their Boltz-2x-reproduced counterparts. The size of the training set in each case is annotated. Details about training set curation are available in the Methods section.

Comparison of AEV-PLIG with Boltz-2 on the FEP benchmark

For the community's interest, we additionally compared the scoring and ranking performance of Boltz-2 with AEV-PLIG and FEP+[2] on the FEP benchmark curated by Ross et al.[3] The AEV-PLIG model used here was trained on HiQBind[4] and BindingNet v1,[5] which represents the strongest model presented in the main text. We report the overall performance and the performance for each target class in Figures S3 and S4, respectively. Overall, the comparison results between AEV-PLIG and FEP+ are consistent with those reported in our

previous study,[6] where the AEV-PLIG model was trained on the combination of PDBbind,[7] BindingNet v1[5] and BindingDB.[8] Among the 11 target classes for which predictions were available from both Boltz-2 and AEV-PLIG, AEV-PLIG outperformed Boltz-2 in 4 (HF2α, BACE1, Renin, and Factor Xa), underperformed in 1 (OX2), and showed statistically indistinguishable performance in the remaining 6 (SYK, PFKB3, p38, CDK8, Galectin, and SHP-2). Methodological details about Boltz-2 binding affinity prediction can be found in the Methods section below.

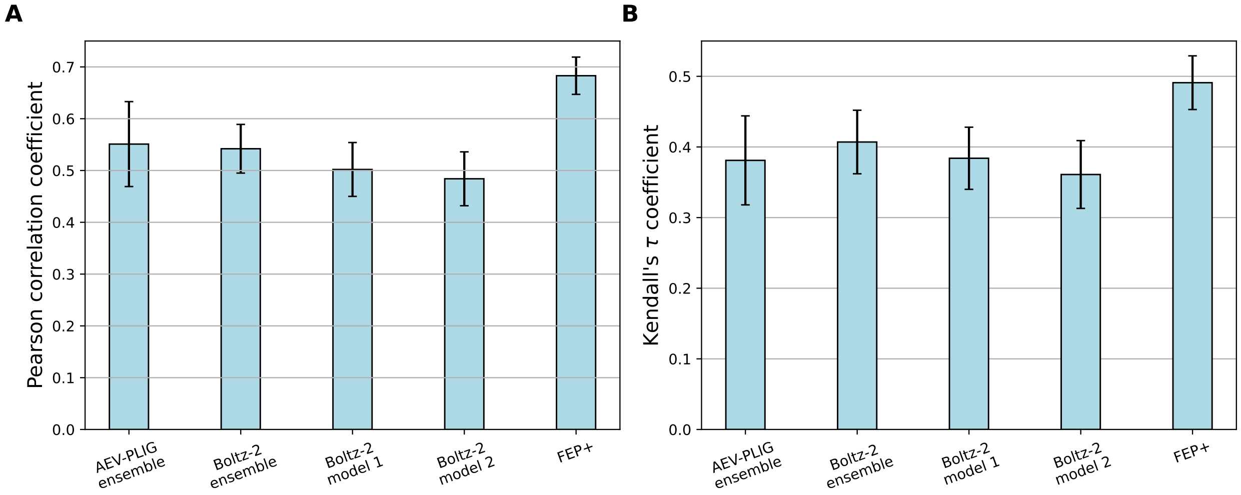

Figure S3: (A) Scoring and (B) ranking performance of Boltz-2, AEV-PLIG, and FEP+ evaluated with the FEP benchmark. Uncertainties are reported as bootstrapped 95% confidence intervals.

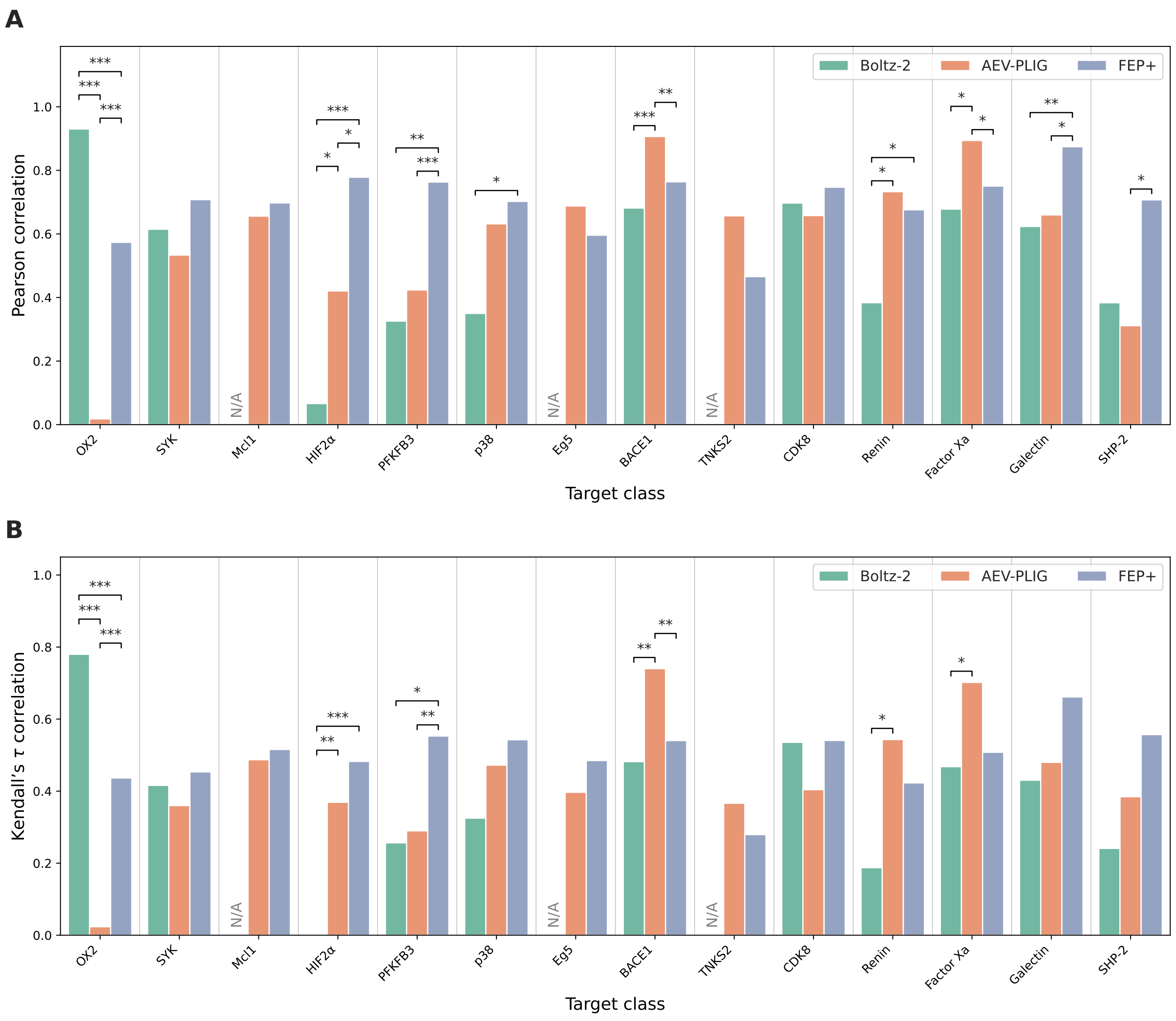

Figure S4: (A) Scoring and (B) ranking performance of Boltz-2 ensemble model, AEV-PLIG ensemble model (trained on HiQBind and BindingNet v1), and FEP+ for each target class in the FEP benchmark. Error bars are omitted for visual clarity. Asterisks denote statistically significant differences between methods (*$p < 0.05$, **$p < 0.01$, ***$p < 0.001$), as determined using the protocol from our previous study.[6] Boltz-2 predictions are unavailable for MCl1, Eg5, and TNKS2 due to out-of-memory errors, which are marked as "N/A" in the figure.

Methods

Model training

For all AEV-PLIG, EHIGN, and RF-Score experiments in this study, we trained an ensemble of five models on NVIDIA A40 GPUs, using the default hyperparameters from their respective original implementations[6,9,10] using NVIDIA A40 GPUs. Complexes that could not be processed by RDKit[11] were discarded, and ligands having uncommon elements (i.e., elements other than H, B, C, N, O, F, P, S, Cl, Br, or I) were excluded. For EHIGN, structures that failed the required PyMOL conversion were also discarded, which affected about 1% of cases. As a result, the FEP benchmark used in EHIGN experiments contained 1167 entries (compared to the original 1184). The number of training examples used in each case is annotated in the relevant figures. RF-Score and AEV-PLIG share the same training sets, while those used for EHIGN are slightly smaller due to PyMOL failures.

To prevent data leakage, any complex whose ligands exhibit a Tanimoto similarity greater than 0.9 to any ligand in the test set (the FEP benchmark) was excluded from the training set. It is worth noting that this choice of similarity cutoff should have a negligible impact on AEV-PLIG training, as the FEP benchmark exhibits minimal chemical overlap with the training sets used. As shown in Figure S5, the vast majority of ligands in HiQBind, BindingNet v1, and BindingNet v2 have low maximum Tanimoto similarity to any ligand in the FEP benchmark.

Binding affinity prediction using scoring functions

All trained scoring functions in this study were tested using the FEP benchmark curated by Ross et al.,[3] which comprises congeneric series commonly seen in real-world drug discovery projects. The dataset covers a wide variety of protein targets and ligands and has minimal overlap with the training sets used in this study, including HiQBind, BindingNet v1, and BindingNet v2. Since most machine learning-based scoring functions (MLSFs) are trained

to predict the negative logarithm of binding affinity (pK), we converted the experimental binding free energy values (ΔG) in the FEP benchmark to pK values using the following equation

$$\Delta G = -\ln(10) RT \mathrm{pK}$$

where the gas constant $R = 1.987 \times 10^{-3}$ kcal $\cdot$ K^{-1} $\cdot$ mol^{-1} and the temperature $T = 297$K. The mean prediction across the ensemble of five models was used for metric calculation. Specifically, we respectively used the Pearson correlation coefficient and Kendall's τ correlation coefficient to assess the scoring power and ranking power of each trained scoring function. All correlation values reported in the main text are weighted averages across FEP benchmark series having ten or more ligands. Uncertainties are reported as bootstrapped 95% confidence intervals.

Binding affinity prediction using Boltz-2

As a supplementary analysis, we compared the scoring and ranking performance of Boltz-2 with AEV-PLIG and FEP+, with results shown in Figures S3 and S4. For each target, the full FASTA sequence was either retrieved from the Protein Data Bank using the available PDB ID or extracted from the ATOM records when metadata was missing. Ligand SMILES strings were generated from the provided SDF files using RDKit.[12] Binding affinity predictions were obtained alongside structure predictions using NVIDIA A40 GPUs with Boltz-2's default inference settings. Predictions were generated using both of the publicly released Boltz-2 models as well as the ensemble model. Of the 1,184 entries in the FEP benchmark, binding affinity predictions were successfully obtained for 969 entries, while the remaining 215 failed due to out-of-memory errors. Consequently, no predictions are available for three target classes, including MCl1, Eg5, and TNKS2, which are indicated as "N/A" in Figure S4. The correlation coefficients are weighted averages across FEP benchmark series having ten or more ligands. Uncertainties are reported as bootstrapped 95% confidence intervals.

Reproduction tasks using Boltz

HiQBind reproduction

HiQBind, recently proposed by Wang et al.,[4] contains 32275 protein-ligand complexes curated from Binding MOAD,[13] BindingDB,[14] and BioLiP,[15] with structural artefacts corrected using a semi-automated computational workflow. For reproduction, we excluded 3469 entries containing non-standard amino acids in the receptor and 82 entries with conformer generation issues from RDKit[11] for the ligand. Additional exclusions were made for entries that failed due to out-of-memory issues during inference or could not be processed by our automatic analysis workflow due to malformed PDB references. The final reproduced dataset contains 28895 predicted structures. For the Boltz-2x reproduction task presented above, the final reproduced dataset contains 29171 entries.

RNP reproduction

Runs and Poses (RNP), recently proposed by Škrinjar et al.,[16] is a benchmark of 2600 experimentally determined protein-ligand complex structures released after the training cutoff date of AF3-like co-folding models. For reproduction, we applied the same filtering criteria used for HiQBind, with an additional restriction to single-chain receptor entries. This ultimately led to a final set of 636 reproduced structures for downstream analysis.

Common settings

All reproduction tasks were performed using NVIDIA A40 GPUs with default settings for Boltz inference, which include 3 recycling steps, 200 sampling steps, a diffusion step size of 1.638, with a greedy MSA pairing strategy. MSA computation was done using the MSA server provided by ColabFold.[17] For entries sharing the same receptor sequence, we reused the same MSA results to accelerate the prediction pipeline. For each binding complex, only one diffusion sample was generated.

Analysis

Boltz provides a wide range of confidence metrics, including pTM, ipTM, ligand ipTM, protein ipTM, complex pLDDT, complex PDE, and complex iPDE, among others. Definitions of these metrics are available in the original technical report.[18] In addition, we compute the DOPE (Discrete Optimised Protein Energy) score[19] to assess the energetic plausibility of the predicted protein structure. Region-specific pLDDT scores are also calculated from the pLDDT matrices provided by Boltz inference, including ligand pLDDT, pocket pLDDT, and shell pLDDT, where pocket and shell residues are defined as those within 0-6 Å and 6-8 Å, respectively, of any ligand heavy atom.

To assess the structural quality of Boltz predictions relative to experimental references, we compute complex RMSD, protein RMSD, ligand RMSD, and pocket RMSD, considering only heavy atoms. For pocket RMSD, the binding pocket is defined based on the reference structure as all residues with any atom within 6 Å of any ligand atom. Structures are aligned using the pocket residues from the reference, and pocket RMSD is then computed over both the pocket residues and the ligand. In our study, a structure is defined as high-quality if it passes the PoseBusters[20] sanity checks and has a pocket RMSD below 2 Å with respect to the reference structure.

Enrichment screening

For investigating the application of the AEV-PLIG models to enrichment screening, three publicly available datasets (D4, AMPC, MPRO) and two in-house datasets from Boehringer Ingelheim Pharma (System A, System B) were considered. The protein-ligand structures (D4: PDBID 5WIU, AMPC: PDBID 6DPX, MPRO: PDBID 6Y2G) were prepared in MOE (Molecular Operating Environment, version 2024.06) using the default settings for protein structure preparation.[21] All crystallographic waters and cofactors were removed. For the publicly available datasets (D4, AMPC, MPRO), ligand SMILES structures were taken from the LSD in vitro dataset information (D4 and AMPC, available at: https://lsd.docking.

org/targets) or the PostEra COVID Moonshot dataset information (MPRO, available at https://covid.postera.ai/covid/activity_data). For the public datasets, actives were defined as the top 5% of the dataset. Ligands were similarly prepared in MOE using the available default settings, with the database of SMILES being converted into 2D molecules, washed, and prepared using the QuickPrep structure preparation. Any ligands that failed the preparation were removed from the dataset. The final numbers of ligands and actives considered are shown in Table S1.

Automated docking was carried out with the GOLD program (Genetic Optimisation for Ligand Docking, version 2025.1.0) using the default parameters, unless otherwise stated.[22] The crystallographic ligand was used to define the binding site cavity and was also used as the reference ligand. The top-ranked generated poses for each molecule were used as input for the AEV-PLIG predictions. Predictions were generated using the models trained on different subsets of the HiQBind and the Boltz-1x-reproduced HiQBind datasets. These include those described in Figure 2, as well as models trained on either high-quality or high-confidence Boltz-1x predictions augmented with the corresponding experimental structures to restore the original dataset size. The 1% enrichment rate of the different models was calculated using a Receiver Operator Characteristic (ROC) curve, and 95% confidence intervals were calculated using stratified bootstrapping with 500 repeats.

Table S1: Compound and active counts for the systems considered for the enrichment screening

**System**	**Number of Compounds**	**Number of Actives**
D4	551	27
AMPC	1490	74
MPRO	1705	102
System A	1431583	6407
System B	1449430	6664

Supplementary Figures

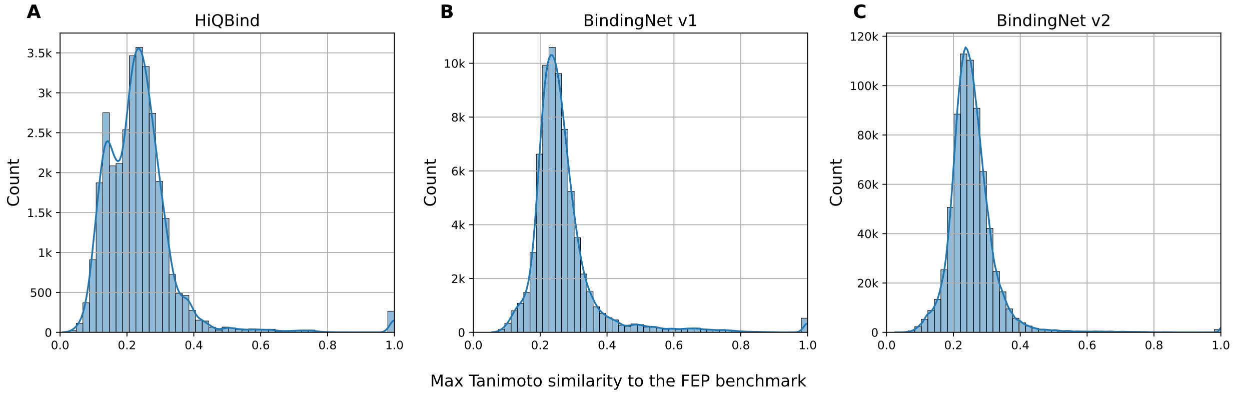

Figure S5: Distribution of maximum Tanimoto similarity between ligands in the FEP benchmark and those in the training sets used in this study: (A) HiQBind, (B) BindingNet v1, and (C) BindingNet v2. All three training sets exhibit minimal ligand overlap with the FEP benchmark, with 97.9%, 96.2%, and 99.1% of entries, respectively, having a maximum similarity below 0.5.

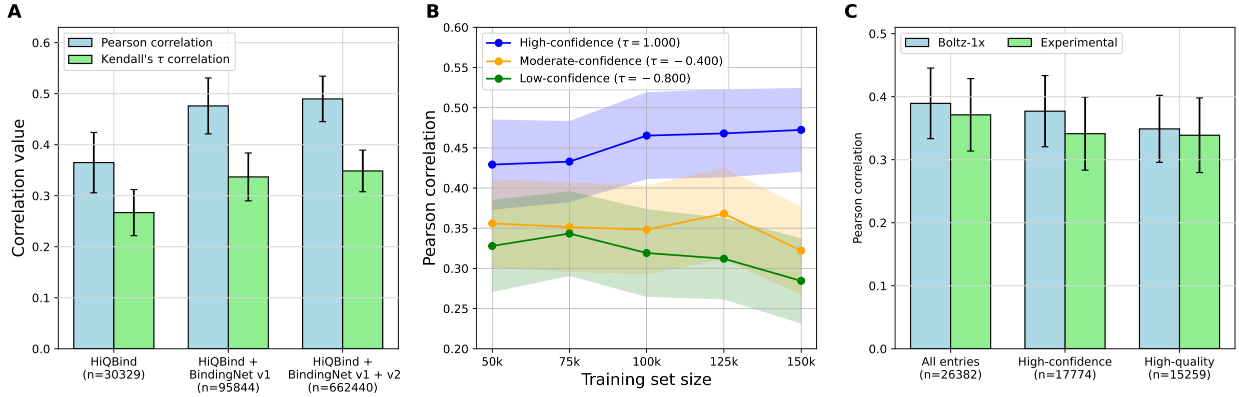

Figure S6: (A) Performance of EHIGNs trained on HiQBind alone, HiQBind + BindingNet v1, and HiQBind + BindingNet v1 + BindingNet v2. The sizes of the datasets are noted in parentheses in the labels. (B) Performance of EHIGNs trained on progressively larger subsets of BindingNet v1 + v2, constructed from different confidence partitions. Each larger subset includes all smaller ones. The Kendall's τ correlations between PCC and training set size for different cases are annotated in the legend. (C) Performance of AEV-PLIGs trained on different subsets of HiQBind and their Boltz-1x-reproduced counterpart. The size of the training set in each case is annotated. Details about training set curation are available in the Methods section.

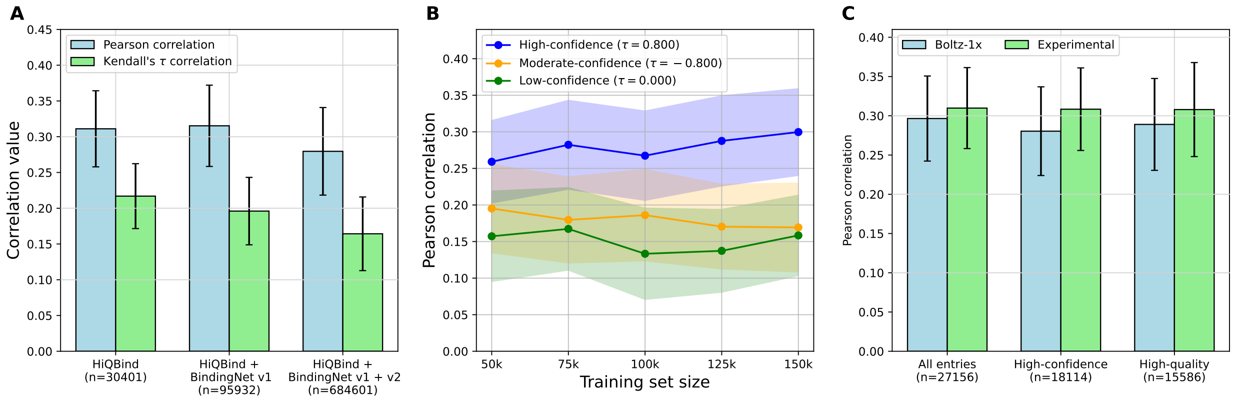

Figure S7: (A) Performance of RF-Scores trained on HiQBind alone, HiQBind + BindingNet v1, and HiQBind + BindingNet v1 + BindingNet v2. The sizes of the datasets are noted in parentheses in the labels. (B) Performance of RF-Scores trained on progressively larger subsets of BindingNet v1 + v2, constructed from different confidence partitions. Each larger subset includes all smaller ones. The Kendall's τ correlations between PCC and training set size for different cases are annotated in the legend. (C) Performance of AEV-PLIGs trained on different subsets of HiQBind and their Boltz-1x-reproduced counterpart. The size of the training set in each case is annotated. Details about training set curation are available in the Methods section.

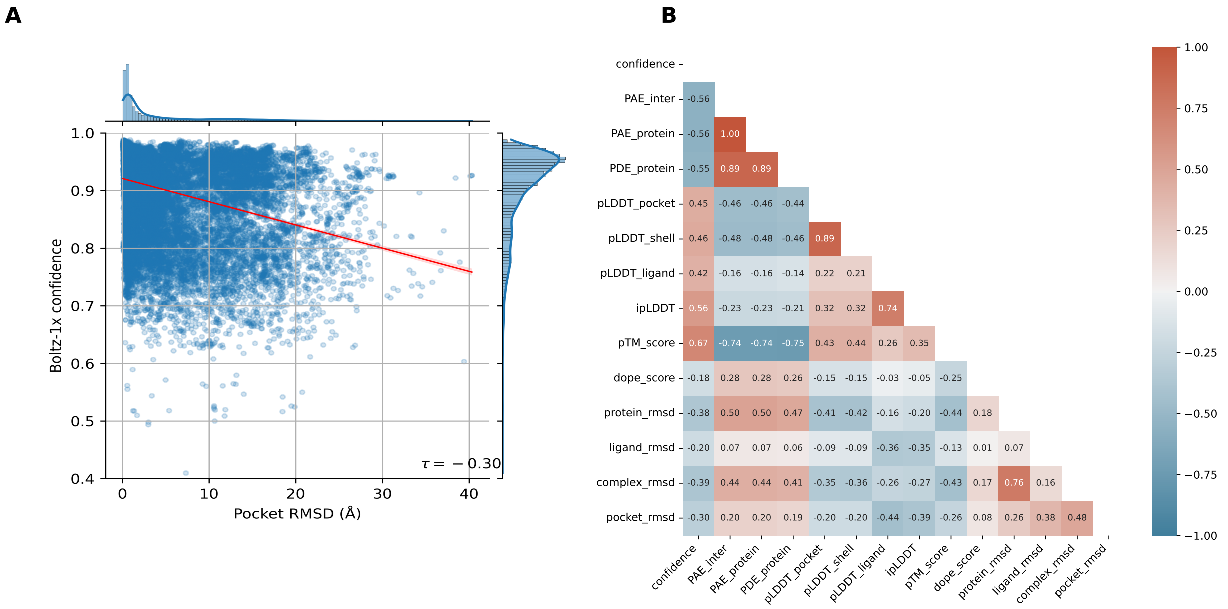

Figure S8: Correlation analysis on Boltz-1x reproduced HiQBind. (A) Scatter plot of Boltz-1x confidence score versus pocket RMSD as a representative example, with the Kendall's τ correlation coefficient annotated. (B) Pairwise Kendall's τ correlation coefficients between commonly used confidence metrics and quality metrics. Both panels show that there is a generally weak confidence-quality correlation in Boltz-1x predictions in the HiQBind reproduction task. More details about how these metrics are defined can be found in the Methods section.

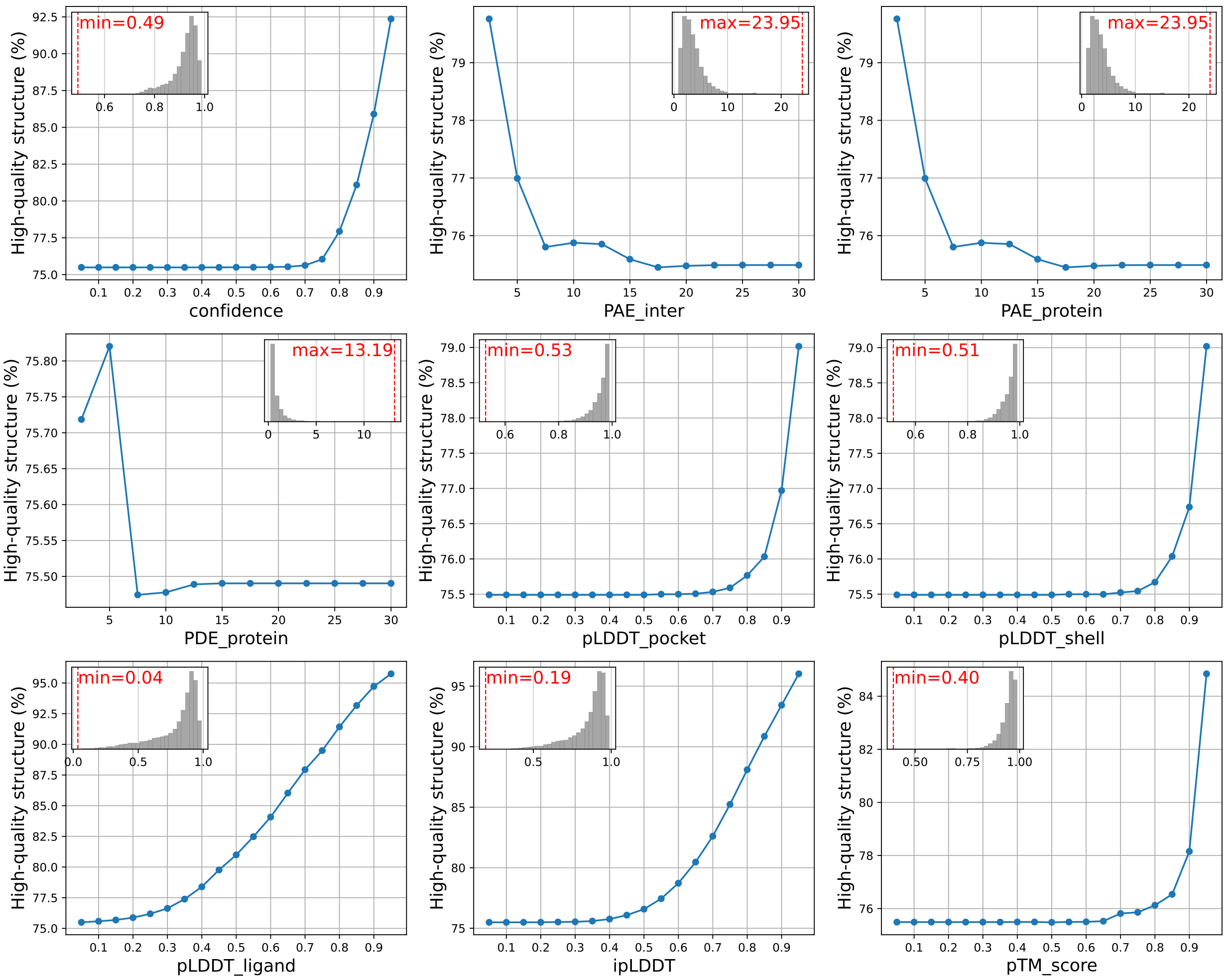

Figure S9: Relationship between confidence metrics and structural accuracy for predicted single-chain, PB-validated entries in the HiQBind reproduction task done using Boltz-1x. Each panel shows the percentage of predicted complexes with pocket RMSD < 2.0 Å as a function of the threshold applied to a given confidence metric. Thresholds are applied in the direction appropriate for each metric, either retaining entries with higher values for metrics where larger values indicate higher confidence, or lower values for those where smaller values indicate higher confidence. Insets show the distribution of the corresponding metric, with a red dashed line indicating either the minimum or maximum value, depending on the direction of thresholding, which corresponds to the least confident prediction according to that metric. For example, the leftmost panel in the first row shows that 86% of the structures having a confidence score higher than 0.9 are of high quality, and the lowest confidence observed in the reproduction task is 0.49.

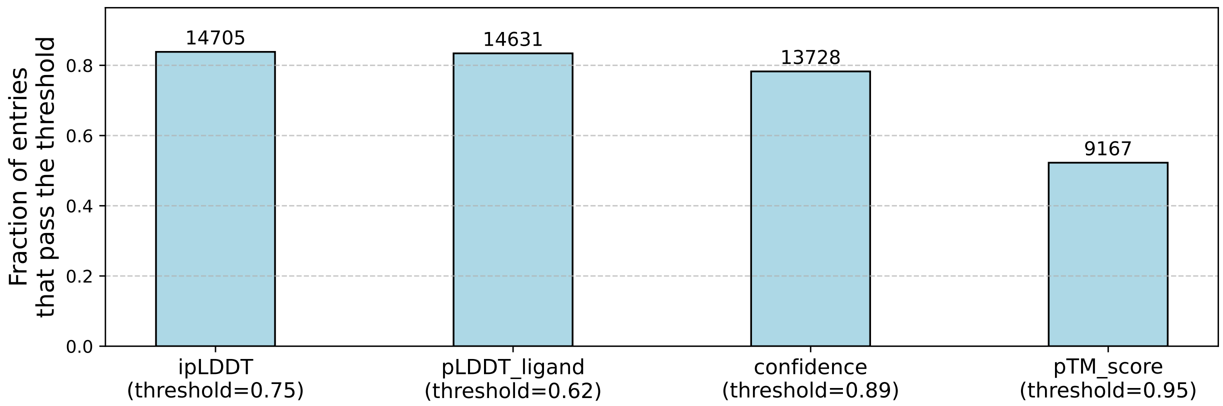

Figure S10: Fraction of entries that pass the threshold required to yield subsets in which at least 85% of predictions are high-quality in the HiQBind reproduction task done using Boltz-1x. Only single-chain, PB-validated predictions are considered here. The number of entries retained at each threshold is annotated above each bar.

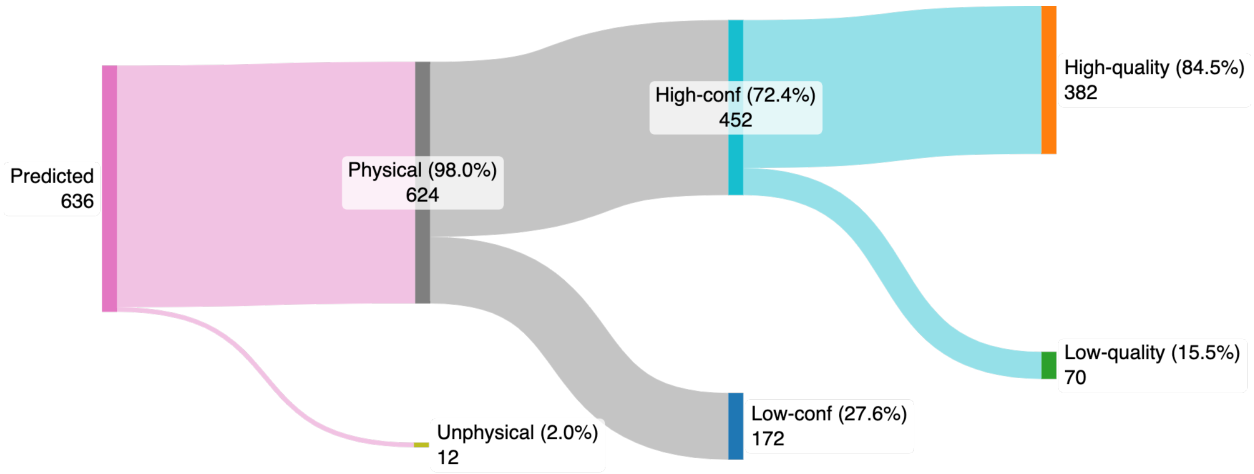

Figure S11: Sankey diagram that summarises the overall performance of Boltz-1x in the RNP reproduction task. Each flow is annotated with the number of predicted structures and its percentage relative to the preceding category. Overall, the proportions of high-confidence predictions and high-quality structures among them are comparable to those observed for single-chain complexes in the HiQBind reproduction task.

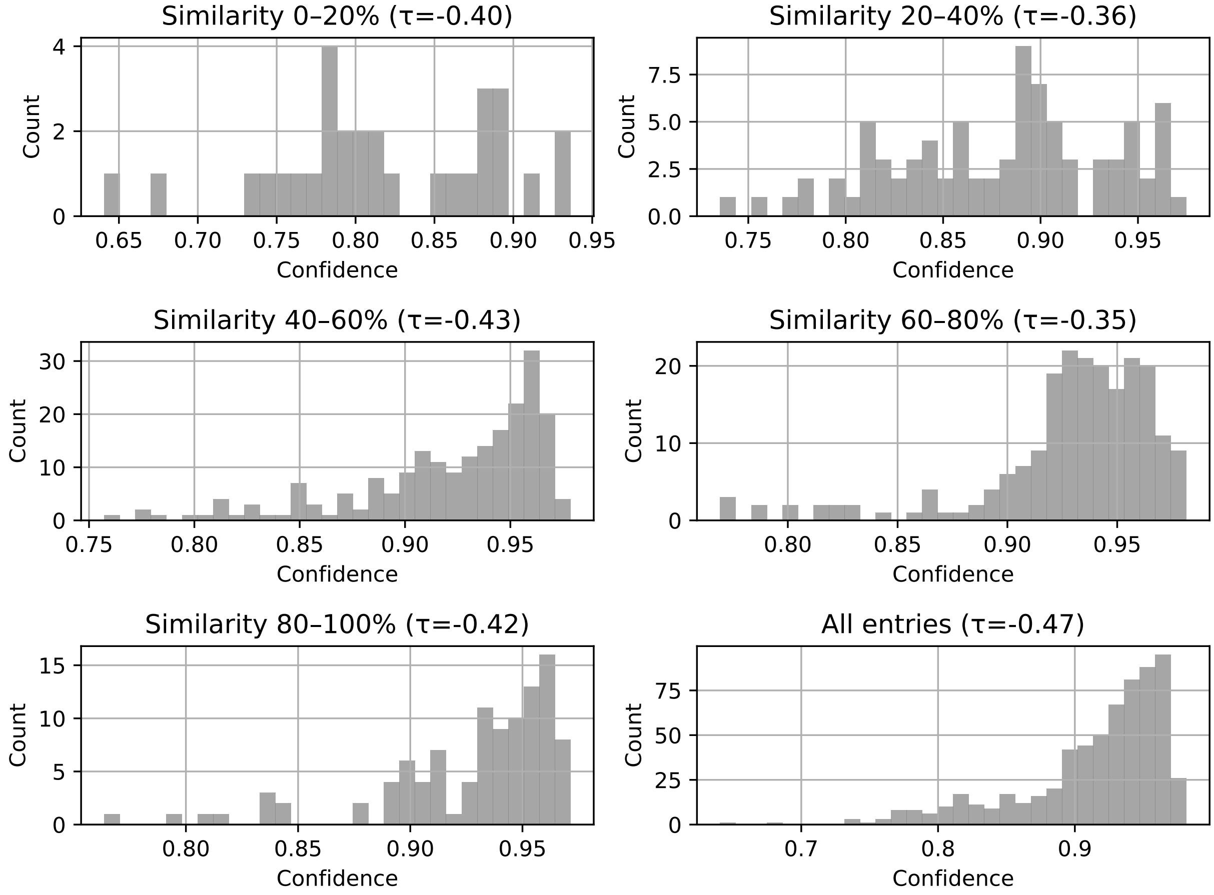

Figure S12: The distributions of Boltz-confidence at different levels of train-test similarity in the RNP reproduction task done using Boltz-1x, with the Kendall's τ correlation between the Boltz confidence and the pocket RMSD annotated. As shown in the figure, this correlation is roughly consistent across all similarity levels. Notably, only single-chain, PB-validated predictions are considered here.

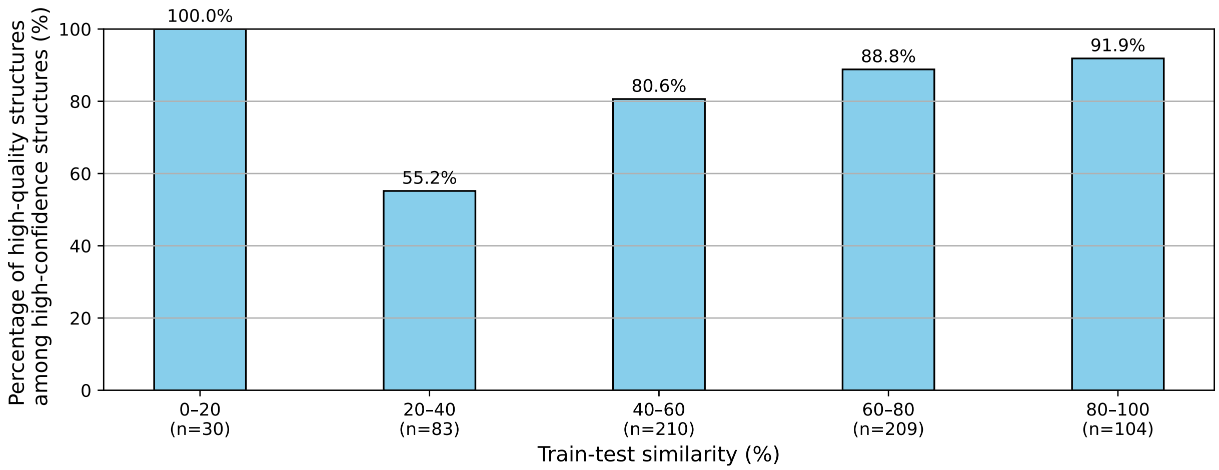

Figure S13: Percentage of high-quality structures within high-confidence subsets at different train-test similarity levels in the RNP reproduction task done using Boltz-1x. Compared to the structure prediction success rate shown in Figure 2B, this trend appears more robust to out-of-distribution (OOD) shifts. We note that the 0–20% and 20–40% similarity bins have fewer samples and may limit statistical confidence.

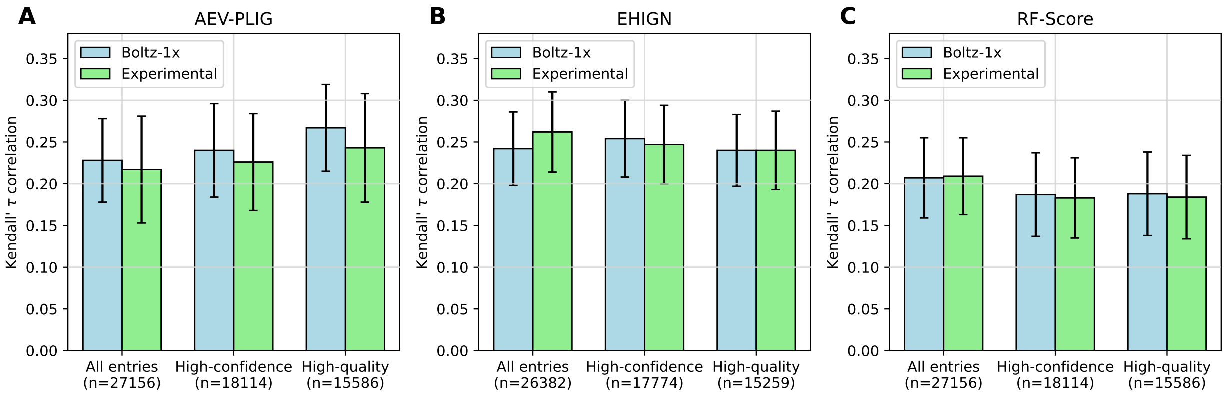

Figure S14: Ranking power of (A) AEV-PLIGs, (B) EHIGNs, and (C) RF-Scores trained on different subsets of HiQBind and their Boltz-1x-reproduced counterpart. The size of the training set in each case is annotated. Details about training set curation are available in the Methods section.

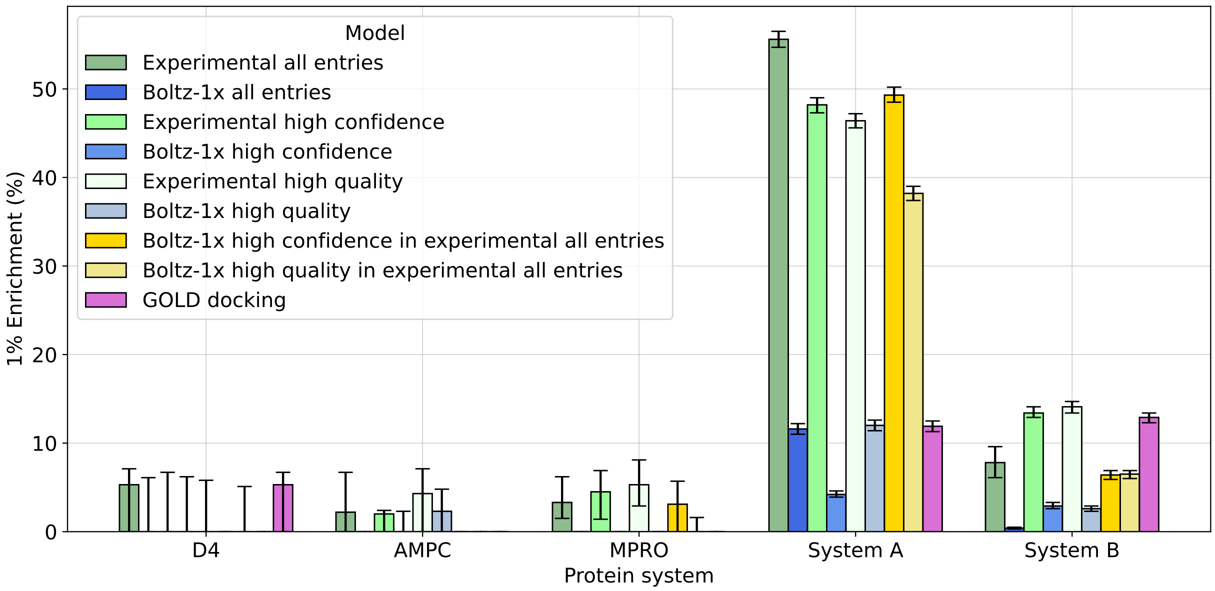

Figure S15: The 1% enrichment rate for the identification of actives for different AEV-PLIG models and protein systems, calculated using a ROC curve. Error bars indicate the 95% confidence intervals from stratified bootstrapping. Overall, the models that were trained on experimental HiQBind data perform better in enrichment tasks than those trained on the Boltz-1x reproduced poses. We note that Systems A and B both have higher ligand similarity with the HiQBind training data and a larger dataset size (Figure S16 and Table S1), which may be responsible for their better performance compared to the public datasets.

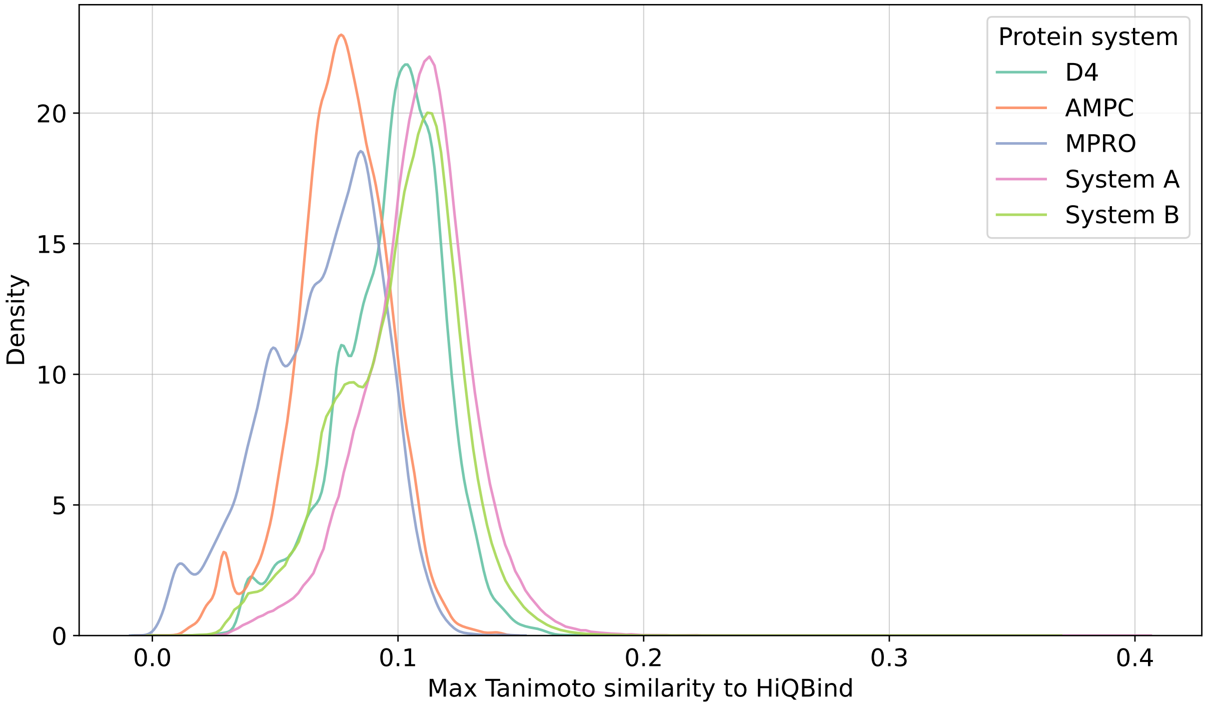

Figure S16: Kernel Density Estimation (KDE) of the Tanimoto ligand similarity to the HiQBind dataset for different protein systems.

References

(1) Passaro, S.; Corso, G.; Wohlwend, J.; Reveiz, M.; Thaler, S.; Ram Somnath, V.; Getz, N.; Portnoi, T.; Roy, J.; Stark, H.; others Boltz-2: Towards Accurate and Efficient Binding Affinity Prediction. *bioRxiv* **2025**, 2025–06.

(2) Abel, R.; Wang, L.; Harder, E. D.; Berne, B.; Friesner, R. A. Advancing drug discovery through enhanced free energy calculations. *Accounts of chemical research* **2017**, *50*, 1625–1632.

(3) Ross, G. A.; Lu, C.; Scarabelli, G.; Albanese, S. K.; Houang, E.; Abel, R.; Harder, E. D.; Wang, L. The maximal and current accuracy of rigorous protein-ligand binding free energy calculations. *Commun. Chem.* **2023**, *6*, 222.

(4) Wang, Y.; Sun, K.; Li, J.; Guan, X.; Zhang, O.; Bagni, D.; Zhang, Y.; Carlson, H. A.; Head-Gordon, T. A workflow to create a high-quality protein–ligand binding dataset for training, validation, and prediction tasks. *Dig. Discov.* **2025**, *4*, 1209–1220.

(5) Li, X.; Shen, C.; Zhu, H.; Yang, Y.; Wang, Q.; Yang, J.; Huang, N. A high-quality data set of protein–ligand binding interactions via comparative complex structure modeling. *J. Chem. Inf. Model.* **2024**, *64*, 2454–2466.

(6) Valsson, Í.; Warren, M. T.; Deane, C. M.; Magarkar, A.; Morris, G. M.; Biggin, P. C. Narrowing the gap between machine learning scoring functions and free energy perturbation using augmented data. *Commun. Chem.* **2025**, *8*, 41.

(7) Wang, R.; Fang, X.; Lu, Y.; Yang, C.-Y.; Wang, S. The PDBbind database: methodologies and updates. *J. Med. Chem.* **2005**, *48*, 4111–4119.

(8) Hu, L.; Benson, M. L.; Smith, R. D.; Lerner, M. G.; Carlson, H. A. Binding MOAD (mother of all databases). *Proteins* **2005**, *60*, 333–340.

(9) Yang, Z.; Zhong, W.; Lv, Q.; Dong, T.; Chen, G.; Chen, C. Y.-C. Interaction-based inductive bias in graph neural networks: enhancing protein-ligand binding affinity predictions from 3d structures. *IEEE Transactions on Pattern Analysis and Machine Intelligence* **2024**, *46*, 8191–8208.

(10) Ballester, P. J.; Mitchell, J. B. A machine learning approach to predicting protein–ligand binding affinity with applications to molecular docking. *Bioinformatics* **2010**, *26*, 1169–1175.

(11) Landrum, G.; others Rdkit: Open-source cheminformatics software. 2016.

(12) Landrum, G.; others Rdkit: Open-source cheminformatics software. 2016.

(13) Hu, L.; Benson, M. L.; Smith, R. D.; Lerner, M. G.; Carlson, H. A. Binding MOAD (mother of all databases). *Proteins* **2005**, *60*, 333–340.

(14) Liu, T.; Lin, Y.; Wen, X.; Jorissen, R. N.; Gilson, M. K. BindingDB: a web-accessible database of experimentally determined protein–ligand binding affinities. *Nucleic Acids Res.* **2007**, *35*, D198–D201.

(15) Yang, J.; Roy, A.; Zhang, Y. BioLiP: a semi-manually curated database for biologically relevant ligand–protein interactions. *Nucleic Acids Res.* **2012**, *41*, D1096–D1103.

(16) Škrinjar, P.; Eberhardt, J.; Durairaj, J.; Schwede, T. Have protein-ligand co-folding methods moved beyond memorisation? *BioRxiv* **2025**, 2025–02.

(17) Mirdita, M.; Schütze, K.; Moriwaki, Y.; Heo, L.; Ovchinnikov, S.; Steinegger, M. ColabFold: making protein folding accessible to all. *Nat. methods* **2022**, *19*, 679–682.

(18) Wohlwend, J.; Corso, G.; Passaro, S.; Reveiz, M.; Leidal, K.; Swiderski, W.; Portnoi, T.; Chinn, I.; Silterra, J.; Jaakkola, T.; others Boltz-1: Democratizing Biomolecular Interaction Modeling. *bioRxiv* **2024**, 2024–11.

(19) Shen, M.-y.; Sali, A. Statistical potential for assessment and prediction of protein structures. *Protein Sci.* **2006**, *15*, 2507–2524.

(20) Buttenschoen, M.; Morris, G. M.; Deane, C. M. PoseBusters: AI-based docking methods fail to generate physically valid poses or generalise to novel sequences. *Chemical Science* **2024**, *15*, 3130–3139.

(21) Chemical Computing Group ULC Molecular Operating Environment (MOE), version 2024.0601. https://www.chemcomp.com, 2025; Software package.

(22) Jones, G.; Willett, P.; Glen, R. C.; Leach, A. R.; Taylor, R. Development and validation of a genetic algorithm for flexible docking11Edited by F. E. Cohen. *Journal of Molecular Biology* **1997**, *267*, 727–748.
